# An international dataset on organic molecule concentrations in soil and related kitchen garden crops

**DOI:** 10.1038/s41597-025-05033-5

**Published:** 2025-05-02

**Authors:** Céline Laurent, Laure Genies, Géraldine Bidar, Elodie-Denise Ripamonti-Chenot, Camille Dumat, Corinne Hulot, Franck Marot, Christophe Schwartz, Stéphanie Ouvrard

**Affiliations:** 1 Université de Lorraine, INRAE, LSE, F-54000 Nancy, France; 2 Université de Lille, Institut Mines-Télécom, Université d’Artois, JUNIA, ULR 4515 - LGCgE, Laboratoire de Génie Civil et géo-Environnement, F-59000 Lille, France; 3 DYNAFOR, INRAE & Toulouse INP, Toulouse, France; 4 Ineris, Parc technologique Alata, BP 2, F-60550 Verneuil-en-Halatte, France; 5 Agence de l’Environnement et de la Maîtrise de l’Energie (ADEME), Direction Villes et Territoires Durables, Services Friches Urbaines et Sites Pollués, 20 Avenue du Grésillé, 49009 Angers, Cedex France

**Keywords:** Environmental impact, Environmental impact

## Abstract

Kitchen garden crops offer many environmental, socio-economic and health benefits but can also result in land use changes that may increase citizens’ exposure to pollutants. In this context, a dataset of organic molecules, based on an analysis of scientific literature was created, entitled BAPPOP (*BAse de données sur la contamination des Plantes Potagères par les molécules Organiques Polluantes*, *i.e*. dataset on contamination of kitchen garden plants by organic pollutants). BAPPOP compiles 6246 entries collected from 87 publications and 3 reports linking data on organic molecule’ concentrations in kitchen garden soil and crops. Additional information relative to the conditions for which the data have been obtained complete the dataset: organic molecule descriptions, soil properties, plant descriptions and treatments, experimental and analytical methods, as well as bibliographic information. This dataset is suitable for various stakeholders involved in environmental analyses both to compare site specific results to scientific literature data and secondly to predictively evaluate organic molecule’ concentrations and their transfer to kitchen garden crops based on the soil concentration.

## Background & Summary

Concomitantly with artificialization^1^ and densification of (sub)urban areas, an emphasis has been placed on kitchen gardens, which offer many benefits for the environment and the population, such as the reintroduction of nature into cities, the creation of social links and the willingness to produce local food and eat more healthily. In towns and cities, the pressure on land and the dynamic toward urban agriculture is such that shared, community or individual gardens are most often set up on areas where the soil is degraded. In France, this trend was reinforced by the Climate and Resilience Act (2021)^2^ requiring neutral balance between artificialization and restoration (zero-net artificialization) by 2050. For example, redeployment of brownfield sites could result in increasing the exposure of citizens to contaminants^3^. Thus, compliance between the soil state and its use in any urban agriculture project needs to be assessed. For this purpose and on the initiative of ADEME (The French Agency for Ecological Transition) and INERIS (French institute of industrial environment and risks), the BAPPOP dataset was created in 2015 and updated in 2023. BAPPOP gathered data on a set of organic molecule concentrations in several kitchen garden crop types and associated soils, in different contexts and origins of contamination. The dataset resulted in a freely available table, located in RechercheDataGouv^4^, an online repository with shared data, conforming to “FAIR” principles. Another dataset^5^, previously created in the same context as BAPPOP, concerning metallic trace elements concentrations in both different kitchen garden crop types and in associated soils (BAPPET dataset, *BAse de données sur la contamination des Plantes Potagères par les Eléments Traces métalliques*, *i.e*. dataset on contamination of kitchen garden plants by metallic trace elements), was also updated in 2023.

The BAPPOP dataset was initially created for people in charge of environmental analyses to compare site-specific results to literature data in identical or similar contexts. A user survey was conducted from December 2022 to February 2023, revealing that the BAPPOP dataset is used either for prediction of soil and/or vegetable concentrations, or for comparison of data with their own specific site data. A research on Web Of Science (WOS) was performed to discern whether the BAPPOP dataset had already been used for scientific analysis, but to our knowledge, this is not yet the case.

## Methods

### Selection of most relevant organic molecules

The first step was to select an appropriate search engine for this collection of bibliographic resources. Three search engines were available at the Université de Lorraine: WOS, Science Direct and Ex Libris. Whether at the creation of BAPPOP in 2015, or when it was updated in 2023, WOS was selected as being the tool returning the highest number of references for a test associating one of the five sub-families of organic pollutants (polycyclic aromatic hydrocarbon-PAH, polychlorinated biphenyls-PCB, polychlorinated dibenzodioxins-PCDD/polychlorinated dibenzofurans-PCDF and benzene, toluene, ethylbenzene and xylene-BTEX) to four target keywords (plant/transfer/uptake/soil).

At the time of the BAPPOP dataset creation in 2015 it appeared essential to define a reasonable list of target molecules to be included in the dataset, so that it would both meet the end-users’ expectations and be adapted to existing literature resources. This list was updated in 2023 following the same decision-making methodology. The decision-making process thus associated three major issues regarding the existence of i) regulations and ii) toxicological reference values, and iii) the availability of published data.

In 2015 a preliminary list of 576 organic molecules was established, based on existing European legislation^6–8^, scientific reports by the European Food Safety and Authority (EFSA 2008^9^ and EFSA 2010^10^) and French monitoring and control plan of animal and plant foodstuffs and animal feedstuffs by the French directorate for food 2004^11^ and 2010^12^. To be further considered, a molecule had to be present in at least three of the above cited regulations, thereby leading in 2015 to a reduced list of 169 molecules. To this list, organic molecules considered as priority substances by environmental and health risk agencies were added on expert opinion. Thus, this led to a list of 235 organic molecules of interest.

Given the way the dataset was to be implemented, it seemed pointless to include molecules which had yet to be studied and for which literature resource was poor or non-existent. A bibliographic criterion, based on the potential availability of data was defined. Using the request in WOS [“plant” AND “transfer” OR “uptake” AND “*name of the organic molecule*”], where “*name of the molecule*” was replaced by the name of one of the 235 pre-selected molecule, initially applied over the period 1950 to June 19^th^ 2014, the *Bibliometric score* set was equal to the number of publications returned by the search +1.

A *Toxicological score* combining hazard and dose scoring was defined as follows. The *Hazard score* was defined according to the French National Institute of Research and Security (INRS) classification on carcinogenic, mutagenic and reprotoxic (CMR) substances^13^ and the directive 1272/2008/CEE^14^ according to the class 1A or 1B, 2 and no CMR classification. If the organic molecule is:

- Class 1A or 1B, the hazard score is equal to 6;
- Class 2, the hazard score is equal to 3;
- Not classified as CMR, the hazard score is equal to 1.

The *Dose score* is given according to the existence of a toxicity reference value (TRV) in the main toxicological databases, like US EPA^15^, ATSDR^16^ and RIVM^17^. A score equal to 2 is attributed to a substance which presents at least one TRV, and a score equal to 1 is attributed when no TRV exists for the substance. The global toxicological score is then given by:

1 $${Toxicological\; score}={Hazard\; score}\ast {Dose\; score}$$

A Global score was then calculated with:

2 $${Global\; score}={Bibliometric\; score}\ast {Toxicological\; score}$$

**Fig. 1:**
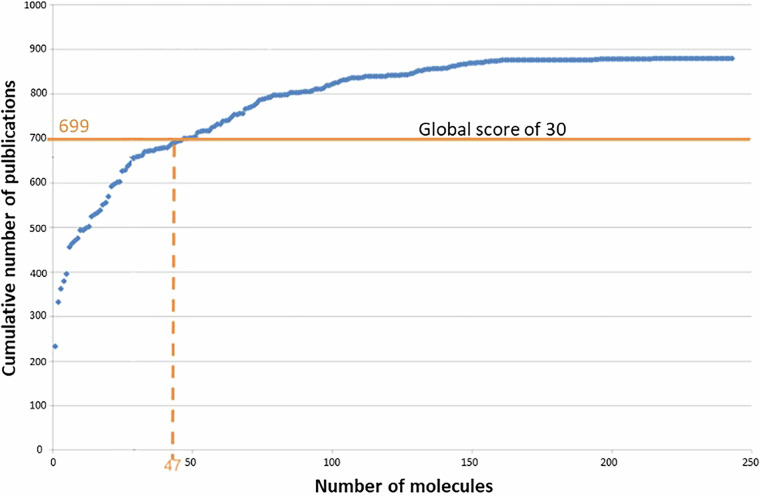
Cumulative number of publications as a function of the number of molecules to consider.

In 2015, this analysis led to the selection of 47 molecules to be considered in the dataset. To represent the quantity of available publications and the number of molecules to consider, the cumulative number of publications as a function of molecule number was represented (Fig. 1). Thus, it makes it possible to assess the gain in publications compared to the number of molecules to be considered. It has been suggested that a threshold of 30 should be set, given that the gain in publishing beyond this is small (Fig. 1). In order not to exclude other non-dioxin like PCB, 7 non-dioxin like PCB were added, resulting in a list of 54 organic molecules of interest.

In 2023, the same methodology was followed, considering the period 2015 to 2022 for the update. Updated legislation was used for the filter application, leading to the addition of 18 new organic molecules to the BAPPOP dataset. Based on the same strategy, 36 molecules that are now prioritised by environmental and risks agencies were added to the BAPPOP dataset update, including PCCD, PCDF and per-and polyfluoroalkyl substances (PFAS), of which 7 PCDD and 10 PCDF that are conventionally assessed in the environment and 19 PFAS based on commonly studied PFAS in scientific literature. Thus, 108 organic molecules of interest are now considered in the BAPPOP dataset.

### Plant selection criteria

**Fig. 2:**
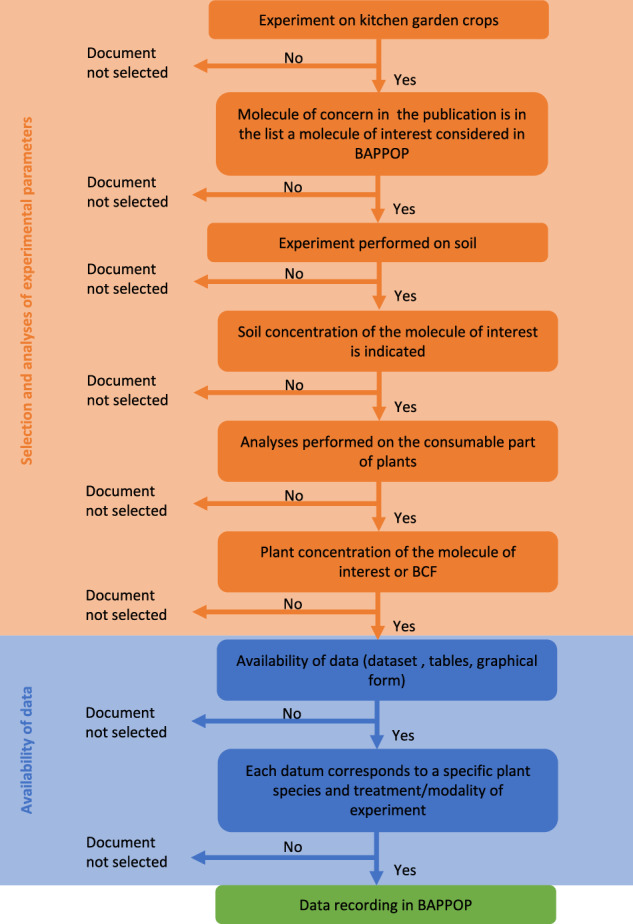
Decision-making flowchart for data implementation in BAPPOP dataset. (BCF stands for bioconcentration factor).

The publications, of whatever origin, from which the data were registered in BAPPOP were selected following a specific decision-making methodology detailed in the Fig. 2 flowchart. The first selection criterion was to refer to experiments on kitchen garden crops exclusively. The kitchen garden crops not cultivated in temperate climates were not included.

With regards to plant species, the consumed organs of plants were taken into account, to the exclusion of all others. For example, a study concerning the analysis of organic molecule concentration in tomato roots was excluded. Similarly, studies involving model plant species (*e.g. Arabidopsis thaliana*) or cereals other than grain of sweet corn were excluded. The molecules studied in publications had to be among the 108 selected molecules of interest. In addition, all experiments in cited studies had to be carried out on soils, leading to the elimination of hydroponic experimentations or direct use of substrates (*e.g*. compost, sewage sludge…) without mixing with soil. Organic molecule concentrations in soils and plants had to be supplied by the publication. Situation in which experimentations were conducted with unrealistically high doses of organic molecules in soils or leading to significant phytotoxic responses of the plant were also excluded. When bioconcentration factors (BCF) were used to express plant uptake, these had to correspond to only one species and one edible part of the plant. Data obtained by averaging the concentration of different plant species or different organs of different plant species or even calculated from models were also excluded.

### Data collection

Relevant international scientific published literature was collected with a search request in WOS database, performed on title, abstract and keywords, using the following equation:

3 $$BAPPOP\,update=(Plant\,\mathrm{AND}\,(Transfer\,OR\,Uptake)\,\mathrm{AND}\, \mbox{``} Name\,of\,the\,organic\,molecule\,of\,interest\mbox{''})$$

At the time of BAPPOP creation in 2015, 1213 occurrences were identified for the period spanning 1950 to June 19^th^ 2014. When updating the dataset in 2022, 2677 occurrences were identified for the subsequent period from 2015 to August 1^st^ 2022. Following the methods described above, 90 references were recorded in BAPPOP with 87 references collected *via* search request on WOS and 3 experimental reports from French institutes.

## Data Records

### Recording of data

The scientific publications and experimental reports are referenced in BAPPOP with DOI when applicable and/or information to make the document findable. A unique source code has been designed to refer to each source document (*e.g*. scientific publication or experimental report) and corresponds to the 3 first letters of the first author’s surname followed by the last two numbers of the publication year of the document.

### BAPPOP structure

**Fig. 3:**
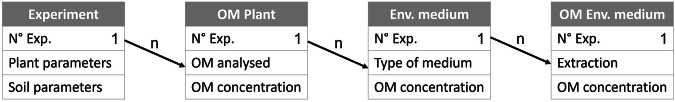
Data structuration in the BAPPOP dataset. Primary keys are in grey. N° refers to the n lines (one or more) for one experiment in the database. Exp: Experiment; OM: Organic molecule; Env. Medium: Environmental medium.

The BAPPOP dataset is organized in 1203 experiments (Fig. 3) collected from 90 bibliographic references on 75 organic molecules, 12 families of organic molecules, 12 plant types and 48 plant species. An experiment is defined as one or a set of organic molecules analysed for one single plant species or variety grown in a single treatment/modality of culture. For each experiment at least the following data are recorded: organic molecule concentration per plant and in the soil supporting their culture. For a given experiment, different environmental media (*i.e*. soil, air, water) may be analyzsd. Thus, in the BAPPOP dataset, there are 6246 recorded observations. The description of the BAPPOP dataset is presented in Table S1.

### Experimental conditions

In the BAPPOP dataset, organic molecules have various pollution origins (*i.e*. agricultural, artificial, industrial, natural, urban or a combination of origins). The environmental context and the experimental types are also detailed when possible, because these parameters can influence the final result.

### Organic molecule parameters

**Fig. 4:**
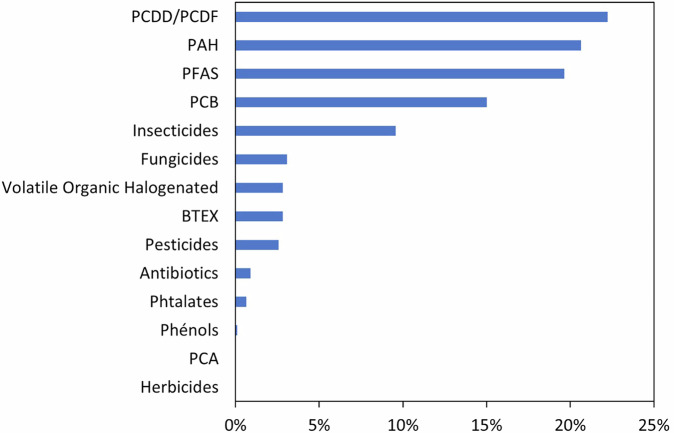
Frequency of observations recorded in the BAPPOP dataset as a function of organic molecule family types.

Each organic molecule is defined by its French and English names and family type, Chemical Abstract Services number (CAS) and chemical formula (Table S1). The list of all the organic molecules considered in BAPPOP is detailed in the BAPPOP metadata associated with the dataset itself in the data repository. The types of organic molecules with the most observations recorded in the BAPPOP dataset are, in decreasing order, PCDD/PCDF, PAH, PFAS and PCB (Fig. 4). It is noteworthy that (i) of the 14 family types of organic molecules considered in the BAPPOP dataset, only 2 families (*i.e*. polychlorinated alkanes-PCA and herbicides) present no observations recorded in the dataset (Fig. 4) and (ii) of the 108 organic molecules considered, only 75 were recorded in the dataset because the other compounds lacked data corresponding to our research criteria. The main criteria for rejection were as follows (i) hydroponic experiments, (ii) the plant species was not considered of interest to increment the BAPPOP dataset, (iii) the concentration of the given organic molecule was not available in both the plant and the soil.

### Plant parameters

**Fig. 5:**
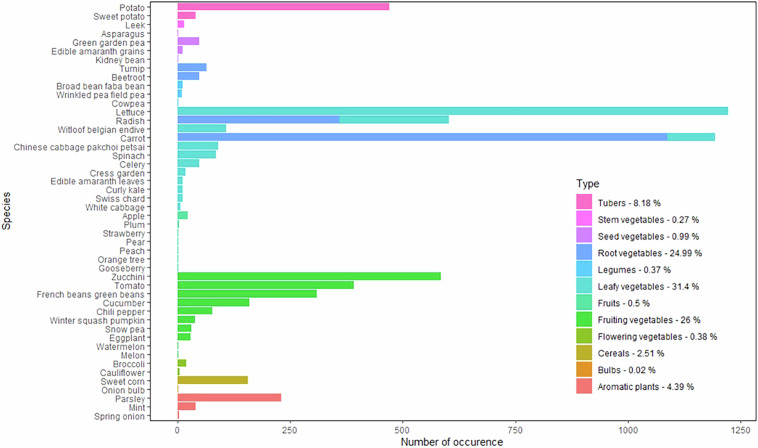
Number of occurrences recorded in the BAPPOP dataset categorized by species and plant types. The proportion of data recorded for each plant type is detailed in the caption.

The BAPPOP dataset describes organic molecule concentration in kitchen garden crops commonly grown in temperate climate conditions. Plant species are classified in 13 family types. It is noteworthy that leafy vegetables are the most represented plant type with 31% of data records, and that no data is available for the “Edible flower” type (Fig. 5, Table S2). Of the 98 plant species considered for inclusion in the BAPPOP dataset (Table S2), only 48 were included due to the lack of research meeting our selection criteria. Carrots and lettuces are the most documented species with over 100 experiments and more than 1000 entries recorded. Other parameters concerning the analyses and the preparation of samples are documented in BAPPOP dataset (Table S1, section “Plant”) whenever available in the source document. No information regarding cultivar and health status (publications reporting phytotoxicity were excluded) of the plant has been implemented.

### Soil parameters

Several parameters known to affect the availability of organic molecules are recorded in the BAPPOP dataset. These parameters concern the soil type which was used as growing support, the soil texture, and classic information about soil chemistry parameters. All details of the 8 parameters considered are given in Table S1 in the “Soil” section.

### Organic molecule analyses in the environmental medium

The BAPPOP dataset focuses on studies reporting organic molecules concentration in soils but, when available, the organic molecule content in water and/or air is also included. The BAPPOP dataset also reports the extraction type and the chemical extractant used to analyse organic molecules (Table S1).

### Data repository

Data collected can be freely accessed through the Recherche Data Gouv repository^4^. The data are organized in .csv files, accompanied by metadata. All variables recorded in the dataset are described in Table S1. To ensure the anonymity of the data, no location is provided concerning the origin in the dataset. Only the country of origin of first author is given when data were collected from scientific literature.

## Technical Validation

The quality control procedure included two points: (i) quality of the data collected, and (ii) quality of the recorded data.

Concerning data collected from scientific literature, it was assumed that the quality of the data was confirmed by the peer-review process. When available, the detection limit and/or quantification limit were recorded in the BAPPOP dataset. Concerning data quality from experimental reports, the studies were supported by French public institutions (ADEME, INERIS) which build various guidelines to optimize experimentation practices^18–20^. Consequently, the use of these guidelines is assumed.

Concerning the quality of recorded data, the operators in charge of BAPPOP incrementation used a Visual Basic Applications (VBA) form with drop-down menus. For numerical inputs, when possible, data were extracted from .pdf files using the import function of Excel. When needed, data were extracted from graphs, using the online software WebPlotDigitizer^21^ with an estimated error between 0 and 16% (mean error 1.7% ± 2.1, median error 0.85%, n = 219 values from 14 different papers).

## Usage Notes

The BAPPOP dataset is suitable (i) to compare site-specific results to scientific literature data, for example as part of an environmental analysis, (ii) to predict organic molecule’ concentration in kitchen garden crops based on the soil concentration and (iii) to identify parameters that may significantly drive the soil to plant transfer of organic molecules. For example, in 2023, a test was performed on the BAPPOP dataset concerning dieldrin, to predict its concentrations in different types of vegetables, based on soil dieldrin concentrations of a residential area. The objective was to assess the suitability of the soil for use as a kitchen garden by the local residents. This revealed that the use of the soil as a kitchen garden did indeed conform to sanitary guidelines for example for growing leafy or root vegetables.

The update by users of the BAPPOP dataset is not possible. Only those individuals responsible for updating BAPPOP dataset are allowed to carry out this task. Giveng the number of bibliographic references added with the update performed in 2023, an update of the BAPPOP dataset around every 5 years seems reasonable.

## Supplementary information

Supplementary tables to

## Data Availability

No custom code has been used.

## References

[1] Béchet, B. *et al*. Sols artificialisés et processus d’artificialisation des sols: Déterminants, impacts et leviers d’action. IFSTTAR et INRA (France), 620 p. (rapport), 127 p. (synthèse). https://www.inrae.fr/sites/default/files/pdf/artificialisation-des-sols-resume-8-p-en-anglais-1.pdf (2017).

[2] Légifrance - Service Public de la diffusion du droit. https://www.legifrance.gouv.fr/jorf/id/JORFTEXT000043956924.

[3] Pelfrêne, A., Sahmer, K., Waterlot, C. & Douay, F. From environmental data acquisition to assessment of gardeners’ exposure: feedback in an urban context highly contaminated with metals. *Environ Sci Pollut Res* **26**, 20107–20120 (2019). 10.1007/s11356-018-3468-y 30353433

[4] Laurent, C. *et al*. BAPPOP – Base de données sur la contamination des plantes potagères par les molécules organiques polluantes. *Entrepôt Recherche Data Gouv*10.57745/MXAU5R (2024).

[5] Genies, L. *et al*. BAPPET - Base de données sur la contamination des plantes potagères par les éléments traces métalliques. *Entrepôt Recherche Data Gouv*10.57745/VERVNL (2024).

[6] European Union. Regulation (EC) No 396/2005 of the European Parliament and of the Council of 23 February 2005 on maximum residue levels of pesticides in or on food and feed of plant and animal origin and amending Council Directive 91/414/EEC Text with EEA relevance. https://eur-lex.europa.eu/legal-content/EN/ALL/?uri=celex%3A32005R0396 (2005).

[7] European Union. Commission Regulation (EC) No 1881/2006 of 19 December 2006 setting maximum levels for certain contaminants in foodstuffs (Text with EEA relevance). https://eur-lex.europa.eu/legal-content/EN/ALL/?uri=CELEX%3A32006R1881 (2006).

[8] European Union. Commission Implementing Regulation (EU) No 788/2012 of 31 August 2012 concerning a coordinated multiannual control programme of the Union for 2013, 2014 and 2015 to ensure compliance with maximum residue levels of pesticides and to assess the consumer exposure to pesticide residues in and on food of plant and animal origin Text with EEA relevance. https://eur-lex.europa.eu/legal-content/EN/TXT/?uri=CELEX%3A32012R0788 (2012).

[9] EFSA (European Food Safety Authority). The 2008 European Union report on pesticides residues in food. EFSA journal 2010; **8** (6): 1646. 442pp. 10.2903/j.efsa.2010.1646 (2010). 10.2903/j.efsa.2010.1646 PMC13085176 42005779

[10] EFSA (European Food Safety Authority). The 2010 European Union report on pesticides residues in food. EFSA journal 2013; 11 (3): 3130. 808pp. 10.2903/j.efsa.2013.3130 (2013). 10.2903/j.efsa.2015.4038 PMC11883121 40061627

[11] Ministère de l’économie des finances et de l’industrie. Direction Générale de la Concurrence, de la Consommation, et de la Répression des Fraudes. Surveillance sanitaire des denrées animales et végétales en France – Bilan 2004 – Plans de surveillance et plans de contrôle (2004).

[12] Ministère de l’économie des finances et de l’industrie. Direction Générale de la Concurrence, de la Consommation, et de la Répression des Fraudes. Surveillance sanitaire des denrées animales et végétales en France – Bilan 2010 – Plans de surveillance et plans de contrôle (2010).

[13] INRS (Institut National de Recherche et de Sécurité), Produits chimiques cancérogènes, mutagènes, toxiques pour la reproduction. Classification réglementaire. ED 976 Aide-mémoire et technique. 98p (2012).

[14] European Union. Commission regulation (EC) No 1272/2008 of the European Parliament and of the Council of 16 December 2008 on classification, labelling and packaging of substances and mixtures, amending and repealing Directives 67/548/EEC and 1999/45/EC, and amending Regulation (EC) No 1907/2006. https://eur-lex.europa.eu/eli/reg/2008/1272/oj/fra (2008).

[15] US-EFA: United States – Environmental Protection Agency - https://iris.epa.gov/AtoZ/?list_type=alpha.

[16] ATSDR: Agency for Toxic Substances and Disease Registry - https://wwwn.cdc.gov/TSP/MRLS/mrlsListing.aspx.

[17] Baars, A. J. *et al*. Re-evaluation of human- toxicological maximum permissible risk levels. RIVM, Rijsinstituut voor volksgezondheid en Milieu. Report 711 701 025 (2001).

[18] ADEME & INERIS. Guide d’échantillonnage des plantes potagères dans le cadre des diagnostics Environnementaux. Seconde édition 67p. (2014).

[19] Damas, O. *et al*. Présomption de pollution d’un sol - Des clés pour comprendre et agir. Plante & Cité, Angers, 36p. (2018).

[20] AgroParisTech, INRAE. Caractérisation de la contamination des sols urbains destinés à la culture maraîchère et évaluation des risques sanitaires. 59 p. (2019).

[21] Rohatgi, A. *WebPlotDigitizer software.* https://automeris.io/WebPlotDigitizer (2022).

