# Supplementary material for: An international dataset on organic molecule concentrations in soil and related kitchen garden crops
Source: Sci Data. 2025 May 2;12:732. doi: 10.1038/s41597-025-05033-5 (PMC12048565; doi:10.1038/s41597-025-05033-5)
Supplement: Supplementary file 1 — Supplementary tables to [file 41597_2025_5033_MOESM1_ESM.docx]

**Supplementary Tables**

Table of Contents

[**Table S1.** Dictionary of the BAPPOP dataset 2](#_Toc188798905)

[**Table S2**. List of plant species and number of data recorded in the BAPPOP database 5](#_Toc188798906)

[**List S1.** List of the publications recorded in the BAPPOP dataset 7](#_Toc188798907)

# **Table S1.** Dictionary of the BAPPOP dataset

| **Topic** | **Variables of the dataset** | **Description** |
| --- | --- | --- |
| Registration in the database | Expérimentation_Experimentation | Number code assigned to a couple of soil-plant data |
|  | Référence_Reference | Code assigned to a source document from scientific literature or from expertise, diagnosis report, etc. This code is composed of the three first letters of first author's name followed by the latest two numbers of the year of publication |
| Organic contaminant | Polluant organique_Organic pollutant (fr), (eng) | Name of the molecule in french (fr) and in english (eng) |
|  | CAS_CAS number | Number of registration of the molecule in the data bank of the Chemical Abtracts Services |
|  | Famille_Family | Type of molecule concerned (*e.g.* herbicids, PFAS, PAH) |
|  | Formule chimique_Chemical formula | Gross chemical formula of the molecule |
|  | Utilisation_Molecule usage | Current use(s) of the molecule |
| Plant | Type plante_Plant type | Type to which the plant belongs to (*e.g.* leafy vegetable, fruit vegetable, root vegetable) |
|  | Espece_Species (fr), (eng), (lat) | Kitchen garden crops considered in BAPPOP are those commonly cultivate in Europe. The name of the plant species is given in french (fr), english (eng) and latin (lat). |
|  | Variété_Variety | Plant species variety when the information is given in the source document |
|  | Nb échantillons_Samples n° | Number of data used to calculate statistics on the concentration of organic pollutant in the plant |
|  | MS/MF _ DW/FW | Expression of concentration in dry (MS) or fresh (MF) biomass |
|  | Moyenne plante_Mean plant, Ecart-type_Standard deviation, Min plante_Min plant, Max plante_Max plant, Médiane_Median | Statistics link with organic pollutant concentration in plant. Values are expressed in mg of pollutant per kg of plant |
|  | BCF, Ecart-type BCF_Standard deviation BCF, BCF min, BCF max | Bioconcentration factor (BCF): ratio between total concentration of organic pollutant in plant (in mg/kg) and total concentration of organic pollutant in soil (in mg/kg). Information about the BCF is given only when this is mentioned in the source document |
|  | Organe_Plant part | The plant organ analysed for measurement of organic pollutant concentration |
|  | Lavage/Pelage_Washing/Peeling | Precision on the plant/organ preparation prior to analyses |
|  | Maturité_Maturity | Information on the plant maturity when it was harvest |
|  | Stade de récolte_Harvesting stage | Harvesting stage of the plant, expressed in days, weeks, months |
|  | Limite de détection plante_Plant detection limit | Detection limit for analysis of organic pollutants in the plants |
|  | Limite de quantification plante_Plant quantification limit | Quantification limit for analysis of organic pollutants in the plants |
| Soil | Sol_soil | Soil type or at least the soil treatment (*e.g.* soil+organic fertilizer) in the experiment, case study, expertise |
|  | Argile (%)_Clay (%) | Percentage of clay in soil |
|  | Sable (%)_Sand (%) | Percentage of sand in soil |
|  | Limon (%)_Silt (%) | Percentage of silt in soil |
|  | pH, pH min and max | Soil pH (meseaured pHwater), minimum and maximum values |
|  | Matiere organique (%)_Organic matter (%) | Percentage of soil organic matter |
|  | C organique (%)_Organic carbon (%) | Percentage of soil organic carbon |
|  | CEC (cmol^+^/kg) | Soil cationic exchange capacity |
| Environment | Milieu_Media | Studied environment (air, soil, water). The unit of expression of concentration in the environmental media is precised in parentheses |
|  | Extraction | Type of extraction performed (total, partial) |
|  | Extractants | Type of chemical substances used to performed organic pollutant extraction from the studied environment samples |
|  | Moyenne_Mean, Ecart-type_Standard deviation, Min, Max, Médiane milieu_Media median | Statistics of organic pollutant concentrations in the studied environment. Units are given in the column "Milieu" |
|  | Seuil détection limite_Dectection limit threshold | Detection limit for analysis of organic pollutant concentration in the environmental media studied |
|  | Limite quantification milieu_Media quantification limit | Quantification limit for analysis of organic pollutant concentration in the environmental media studied |
|  | Prélèvement du sol_Soil sampling | Time when the soil was collected (*e.g.* before plant harvest, concomitantly to plant harvest…) |
| Contamination | Type expérimental_Experiment type | Type of experiment from which the data are derived. This make it possible to distinguish between growing conditions referred to in BAPPOP as "outdoor" (agricutlural field, kitchen garden, container) and controlled growing conditions referred to in BAPPOP as "indoor" (greenhouse, phytotron) |
|  | Contexte_Background | Environmental context of the experimentation. There are industrial, urban and rural contexts, that can be combined |
|  | Origine_Origin | Origin of organic pollutant. There are industrial, natural, urban, and artifical origins |
|  | Commentaire Pollution_Comments | Complementary information on the environmental context, the organic pollutant origin, experimental treatments... |
| Publication source | Auteur1, auteur2_Author, Titre Article_Article title, Année_Year, Journal, Volume, Numéro, Pages, DOI | Source document infomation on authors, title, year of publication, the journal in which the study was published, the volume, number of the journal, number of pages, and the DOI that allowed users of the database to find the referenced article. |
|  | Pays_Country | Country from the first authorss' research organism |
|  | Nature | Nature of the source document, *e.g.* scientific journal, expertise report, environmental diagnosis… |

# **Table S2**. List of plant species and number of data recorded in the BAPPOP database

| **Plant type** | **Plant species** | **Number of data recorded in the BAPPOP database** |
| --- | --- | --- |
| Fruits | Apple | 23 |
| Fruits | Apricot | 0 |
| Leafy vegetables | Arugula, rocket | 0 |
| Stem vegetables | Asparagus | 2 |
| Aromatic plants | Basil, great basil | 0 |
| Root vegetables | Beetroot | 49 |
| Fruits | Blackberry | 0 |
| Fruits | Blackcurrant | 0 |
| Legumes | Broad bean, faba bean | 11 |
| Flowering vegetables | Broccoli | 19 |
| Leafy vegetables | Brussels sprouts | 0 |
| Fruiting vegetables | Calbash, bottle gourd | 0 |
| Stem vegetables | Cardoon | 0 |
| Leafy vegetables | Carrot | 105 |
| Root vegetables | Carrot | 1088 |
| Flowering vegetables | Cauliflower | 5 |
| Leafy vegetables | Celery | 49 |
| Root vegetables | Celriac | 0 |
| Fruits | Cherry | 0 |
| Aromatic plants | Chervil | 0 |
| Legumes | Chickpea | 0 |
| Fruiting vegetables | Chili, pepper | 78 |
| Leafy vegetables | Chinese cabbage, pakchoi, petsai | 90 |
| Aromatic plants | Chives | 0 |
| Fruits | Citron | 0 |
| Aromatic plants | Coriander | 0 |
| Legumes | Cowpea | 2 |
| Leafy vegetables | Cress/garden cress/peppergrass | 17 |
| Fruiting vegetables | Cucumber (and gherkin) | 160 |
| Leafy vegetables | Curled-leaved endive, broad-leaved endive | 0 |
| Leafy vegetables | Curly kale | 12 |
| Seed vegetables | Edible amaranth (grains) | 12 |
| Leafy vegetables | Edible amaranth (leaves) | 12 |
| Fruiting vegetables | Eggplant | 29 |
| Leafy vegetables | Fennel | 0 |
| Bulbs | French shallot | 0 |
| Fruiting vegetables | French beans, green beans | 309 |
| Aromatic plants | Garden angelica, wild celery | 0 |
| Edible flowers | Garden nasturtium | 0 |
| Bulbs | Garlic | 0 |
| Flowering vegetables | Globe artichoke | 0 |
| Fruits | Gooseberry | 1 |
| Fruits | Grape, grapevine | 0 |
| Seed vegetables | Green or garden pea | 48 |
| Root vegetables | Horseradish | 0 |
| Aromatic plants | Japanese bunching onion | 0 |
| Seed vegetables | Kidney bean | 2 |
| Fruits | KiwiFruits, kiwi, chinese gooseberry | 0 |
| Stem vegetables | Kohlrabi | 0 |
| Fruits | Kumquat | 0 |
| Leafy vegetables | Land cress, winter cress | 0 |
| Stem vegetables | Leek | 15 |
| Fruits | Lemon | 0 |
| Aromatic plants | Lemon balm | 0 |
| Leafy vegetables | Lettuce | 1222 |
| Leafy vegetables | Mache, cornsalad, lamb's lettuce | 0 |
| Fruits | Mandarin | 0 |
| Aromatic plants | Marjoram | 0 |
| Fruiting vegetables | Melon | 2 |
| Aromatic plants | Mint | 41 |
| Fruiting vegetables | Olive | 0 |
| Bulbs | Onion (bulb) | 1 |
| Aromatic plants | Onion (leaves), spring onion | 3 |
| Fruits | Orange tree | 1 |
| Aromatic plants | Parsley | 230 |
| Root vegetables | Parsnip | 0 |
| Fruits | Peach | 1 |
| Fruits | Pear | 1 |
| Fruits | Plum | 3 |
| Fruits | Pomelo | 0 |
| Tubers | Potato | 471 |
| Fruits | Quince | 0 |
| Leafy vegetables | Radish | 243 |
| Root vegetables | Radish | 359 |
| Fruits | Raspberry | 0 |
| Leafy vegetables | Red cabbage | 0 |
| Leafy vegetables | Rhubarb | 0 |
| Root vegetables | Rutabaga or yellow turnip | 0 |
| Aromatic plants | Sage | 0 |
| Aromatic plants | Savory | 0 |
| Root vegetables | Scorzonera, black salsify, spanish salsify | 0 |
| Fruiting vegetables | Snow pea | 30 |
| Leafy vegetables | Sorrel, garden sorrel | 0 |
| Fruits | Sour cherry | 0 |
| Leafy vegetables | Spinach | 85 |
| Fruits | Strawberry | 1 |
| Cereals | Sweet corn | 157 |
| Tubers | Sweet potato | 40 |
| Leafy vegetables | Swiss chard | 11 |
| Aromatic plants | Tarragon, estragon | 0 |
| Aromatic plants | Thyme | 0 |
| Fruiting vegetables | Tomato | 391 |
| Tubers | Topinanbur, Jerusalem artichoke | 0 |
| Root vegetables | Turnip | 65 |
| Fruiting vegetables | Watermelon | 2 |
| Leafy vegetables | White cabbage | 7 |
| Fruiting vegetables | Winter squash, pumpkin | 38 |
| Leafy vegetables | Witloof, belgian endive | 108 |
| Legumes | Wrinkled pea, field pea | 10 |
| Fruiting vegetables | Zucchini or marrow | 585 |

# **List S1.** List of the publications recorded in the BAPPOP dataset

1. Abhilash, P. C., Jamila, S., Singh, V., Singh, A., Singh, N. & Srivastava, S. C. Occurrence and distribution of hexachlorocyclohexane isomers in vegetation samples from a contaminated area. Chemosphere 72, 79–86 (2008).

2. Abril, C., Santos, J. L., Martín, J., Aparicio, I. & Alonso, E. Uptake and translocation of multiresidue industrial and household contaminants in radish grown under controlled conditions. Chemosphere 268, 128823 (2021).

3. Affholder, M.-C., Cohen, G. J. V., Gombert-Courvoisier, S. & Mench, M. Inter and intraspecific variability of dieldrin accumulation in Cucurbita fruits: New perspectives for food safety and phytomanagement of contaminated soils. Science of The Total Environment 160152 (2022). doi:10.1016/j.scitotenv.2022.160152

4. Al Nasir, F. & Batarseh, M. I. Agricultural reuse of reclaimed water and uptake of organic compounds: Pilot study at Mutah University wastewater treatment plant, Jordan. Chemosphere 72, 1203–1214 (2008).

5. Ansari, S., Waheed, S., Ali, U., Jones, K. C., Sweetman, A. J., Halsall, C. & Malik, R. N. Assessing residual status and spatial variation of current-use pesticides under the influence of environmental factors in major cash crop growing areas of Pakistan. Chemosphere 212, 486–496 (2018).

6. Bizkarguenaga, E., Zabaleta, I., Prieto, A., Fernández, L. A. & Zuloaga, O. Uptake of 8:2 perfluoroalkyl phosphate diester and its degradation products by carrot and lettuce from compost-amended soil. Chemosphere 152, 309–317 (2016).

7. Bizkarguenaga, E., Zabaleta, I., Mijangos, L., Iparraguirre, A., Fernández, L. A., Prieto, A. & Zuloaga, O. Uptake of perfluorooctanoic acid, perfluorooctane sulfonate and perfluorooctane sulfonamide by carrot and lettuce from compost amended soil. Science of The Total Environment 571, 444–451 (2016).

8. Blaine, A. C., Rich, C. D., Hundal, L. S., Lau, C., Mills, M. A., Harris, K. M. & Higgins, C. P. Uptake of Perfluoroalkyl Acids into Edible Crops via Land Applied Biosolids: Field and Greenhouse Studies. Environ. Sci. Technol. 47, 14062–14069 (2013).

9. Blaine, A. C., Rich, C. D., Sedlacko, E. M., Hundal, L. S., Kumar, K., Lau, C., Mills, M. A., Harris, K. M. & Higgins, C. P. Perfluoroalkyl Acid Distribution in Various Plant Compartments of Edible Crops Grown in Biosolids-Amended soils. Environ. Sci. Technol. 48, 7858–7865 (2014).

10. Bogolte, B. T., Ehlers, G. A. C., Braun, R. & Loibner, A. P. Estimation of PAH bioavailability to Lepidium sativum using sequential supercritical fluid extraction – a case study with industrial contaminated soils. European Journal of Soil Biology 43, 242–250 (2007).

11. Boskovic, N., Bilkova, Z., Sudoma, M., Bielska, L., Skulcova, L., Ribitsch, D., Soja, G., Vrana, B. & Hofman, J. Effects of biochar on the fate of conazole fungicides in soils and their bioavailability to earthworms and plants. Environ. Sci. Pollut. Res. 29, 23323–23337 (2022).

12. Cabidoche, Y. M. & Lesueur-jannoyer, M. Contamination of Harvested Organs in Root Crops Grown on Chlordecone-Polluted Soils. Pedosphere 22, 562–571 (2012).

13. CARTHAGE : Contribution de l’air dans les risques et transferts associés aux HAP en agricultures urbaines. La librairie ADEME at <https://librairie.ademe.fr/air/7650-carthage-contribution-de-l-air-dans-les-risques-et-transferts-associes-aux-hap-en-agricultures-urbaines.html>

14. Chen, H., Yang, X., Gielen, G., Mandal, S., Xu, S., Guo, J., Shaheen, S. M., Rinklebe, J., Che, L. & Wang, H. Effect of biochars on the bioavailability of cadmium and di-(2-ethylhexyl) phthalate to Brassica chinensis L in contaminated soils. Sci. Total Environ. 678, 43–52 (2019).

15. Choi, G.-H., Lee, D.-Y., Seo, D.-C., Kim, L., Lim, S.-J., Ryu, S.-H., Park, B.-J., Kim, J.-H. & Kim, J. H. Endosulfan Plant Uptake Suppression Effect on Char Amendment in Oriental Radish. Water Air Soil Pollut. 229, 24 (2018).

16. Choi, G.-H., Lee, D.-Y., Bruce-Vanderpuije, P., Song, A.-R., Lee, H.-S., Park, S.-W., Lee, J.-H., Megson, D. & Kim, J.-H. Environmental and dietary exposure of perfluorooctanoic acid and perfluorooctanesulfonic acid in the Nakdong River, Korea. Environ Geochem Health 43, 347–360 (2021).

17. Clostre, F., Letourmy, P., Turpin, B., Carles, C. & Lesueur-Jannoyer, M. Soil Type and Growing Conditions Influence Uptake and Translocation of Organochlorine (Chlordecone) by Cucurbitaceae Species. Water Air Soil Pollut 225, 2153 (2014).

18. Clostre, F., Letourmy, P., Thuriès, L. & Lesueur-Jannoyer, M. Effect of home food processing on chlordecone (organochlorine) content in vegetables. Science of The Total Environment 490, 1044–1050 (2014).

19. Clostre, F., Philippe, L. & Magalie, L.-J. Organochlorine (chlordecone) uptake by root vegetables. Chemosphere 118, 96–102 (2015).

20. Dalahmeh, S., Tirgani, S., Komakech, A. J., Niwagaba, C. B. & Ahrens, L. Per- and polyfluoroalkyl substances (PFASs) in water, soil and plants in wetlands and agricultural areas in Kampala, Uganda. Science of The Total Environment 631–632, 660–667 (2018).

21. McDonough, A. M., Bird, A. W., Freeman, L. M., Luciani, M. A. & Todd, A. K. Fate and budget of poly- and perfluoroalkyl substances in three common garden plants after experimental additions with contaminated river water. Environmental Pollution 285, 117115 (2021).

22. Felizeter, S., Jürling, H., Kotthoff, M., De Voogt, P. & McLachlan, M. S. Influence of soil on the uptake of perfluoroalkyl acids by lettuce: A comparison between a hydroponic study and a field study. Chemosphere 260, 127608 (2020).

23. Felizeter, S., Jürling, H., Kotthoff, M., De Voogt, P. & McLachlan, M. S. Uptake of perfluorinated alkyl acids by crops: results from a field study. Environ. Sci.: Processes Impacts 23, 1158–1170 (2021).

24. Fojtova, D., Vasickova, J., Grillo, R., Bilkova, Z., Simek, Z., Neuwirthova, N., Kah, M. & Hofman, J. Nanoformulations can significantly affect pesticide degradation and uptake by earthworms and plants. Environ. Chem. 16, 470–481 (2019).

25. Gao, Y. & Zhu, L. Plant uptake, accumulation and translocation of phenanthrene and pyrene in soils. Chemosphere 55, 1169–1178 (2004).

26.Gao, C., Hua, Z. & Li, X. Distribution, sources, and dietetic-related health risk assessment of perfluoroalkyl acids (PFAAs) in the agricultural environment of an industrial-agricultural interaction region (IAIR), Changshu, East China. Science of The Total Environment 809, 152159 (2022).

27. Gaw, S. K., Kim, N. D., Northcott, G. L., Wilkins, A. L. & Robinson, G. Uptake of ΣDDT, Arsenic, Cadmium, Copper, and Lead by Lettuce and Radish Grown in Contaminated Horticultural Soils. J. Agric. Food Chem. 56, 6584–6593 (2008).

28. Gonzalez, M., Miglioranza, K. S. B., de Moreno, J. E. A. & Moreno, V. J. Occurrence and distribution of organochlorine pesticides (OCPs) in tomato (Lycopersicon esculentum) crops from organic production. J. Agric. Food Chem. 51, 1353–1359 (2003).

29. Gonzalez, M., Miglioranza, K. S. B., de Moreno, J. E. A. & Moreno, V. J. Organochlorine pesticide residues in leek (Allium porrum) crops grown on untreated soils from an agricultural environment. J. Agric. Food Chem. 51, 5024–5029 (2003).

30. Gonzalez, M., Miglioranza, K. S. B., Aizpún De Moreno, J. E. & Moreno, V. J. Evaluation of conventionally and organically produced vegetables for high lipophilic organochlorine pesticide (OCP) residues. Food and Chemical Toxicology 43, 261–269 (2005).

31. Gredelj, A., Nicoletto, C., Valsecchi, S., Ferrario, C., Polesello, S., Lava, R., Zanon, F., Barausse, A., Palmeri, L., Guidolin, L. & Bonato, M. Uptake and translocation of perfluoroalkyl acids (PFAA) in red chicory (Cichorium intybus L.) under various treatments with pre-contaminated soil and irrigation water. Science of The Total Environment 708, 134766 (2020).

32. Haller, H., Jonsson, A., Lacayo Romero, M. & Jarquín Pascua, M. Bioaccumulation and translocation of field-weathered toxaphene and other persistent organic pollutants in three cultivars of amaranth (A. cruentus ‘R127 México’, A. cruentus ‘Don León’ y A. caudatus ‘CAC 48 Perú’) – A field study from former cotton fields in Chinandega, Nicaragua. Ecological Engineering 121, 65–71 (2018).

33. Hashimoto, Y. Reduction of dieldrin concentration in cucumber fruits using Cucurbita rootstocks and activated carbon. Journal of Pesticide Science 32, 229–234 (2007).

34. Hilber, I., Mäder, P., Schulin, R. & Wyss, G. S. Survey of organochlorine pesticides in horticultural soils and there grown Cucurbitaceae. Chemosphere 73, 954–961 (2008).

35. Huelster, Anke., Mueller, J. F. & Marschner, Horst. Soil-Plant Transfer of Polychlorinated Dibenzo-p-dioxins and Dibenzofurans to Vegetables of the Cucumber Family (Cucurbitaceae). Environ. Sci. Technol. 28, 1110–1115 (1994).

36. Hurtado, C., Canameras, N., Dominguez, C., Price, G. W., Comas, J. & Bayona, J. M. Effect of soil biochar concentration on the mitigation of emerging organic contaminant uptake in lettuce. J. Hazard. Mater. 323, 386–393 (2017).

37. Hwang, J.-I., Lee, S.-E. & Kim, J.-E. Plant Uptake and Distribution of Endosulfan and Its Sulfate Metabolite Persisted in Soil. PLoS One 10, e0141728 (2015).

38. Hwang, J.-I., Lee, S.-E. & Kim, J.-E. Comparison of theoretical and experimental values for plant uptake of pesticide from soil. PLoS One 12, e0172254 (2017).

39. Hwang, K.-W., Yoo, S. C., Lee, S.-E. & Moon, J.-K. Residual Level of Chlorpyrifos in Lettuces Grown on Chlorpyrifos-Treated Soils. Appl. Sci.-Basel 8, 2343 (2018).

40. Hwang, J.-I., Zimmerman, A. R. & Kim, J.-E. Bioconcentration factor-based management of soil pesticide residues: Endosulfan uptake by carrot and potato plants. Sci. Total Environ. 627, 514–522 (2018).

41. Hwang, K.-W. & Moon, J.-K. Translocation of chlorpyrifos residue from soil to Korean cabbage. Appl. Biol. Chem. 61, 145–152 (2018).

42. Hwang, J.-I. & Kim, J.-E. Uptake of endosulfan isomers from soils by leafy vegetable lettuce: A comparative study between model-predicted and field-experimented results. Sci. Total Environ. 844, 157056 (2022).

43. Khan, S., Waqas, M., Ding, F., Shamshad, I., Arp, H. P. H. & Li, G. The influence of various biochars on the bioaccessibility and bioaccumulation of PAHs and potentially toxic elements to turnips (Brassica rapa L.). Journal of Hazardous Materials 300, 243–253 (2015).

44. Khan, K. Y., Ali, B., Zhang, S., Stoffella, P. J., Yuan, S., Xia, Q., Qu, H., Shi, Y., Cui, X. & Guo, Y. Effects of antibiotics stress on growth variables, ultrastructure, and metabolite pattern of Brassica rapa ssp. chinensis. Sci. Total Environ. 778, 146333 (2021).

45. Kumar, V., Sood, C., Jaggi, S., Ravindranath, S. D., Bhardwaj, S. P. & Shanker, A. Dissipation behavior of propargite––an acaricide residues in soil, apple (Malus pumila) and tea (Camellia sinensis). Chemosphere 58, 837–843 (2005).

46. Lal, M. S., Megharaj, M., Naidu, R. & Bahar, M. M. Uptake of perfluorooctane sulfonate (PFOS) by common home-grown vegetable plants and potential risks to human health. Environmental Technology & Innovation 19, 100863 (2020).

47. Lasee, S., Subbiah, S., Thompson, W. A., Karnjanapiboonwong, A., Jordan, J., Payton, P. & Anderson, T. A. Plant Uptake of Per- and Polyfluoroalkyl Acids under a Maximum Bioavailability Scenario. Environmental Toxicology and Chemistry 38, 2497–2502 (2019).

48. Lasee, S., Subbiah, S., Deb, S., Karnjanapiboonwong, A., Payton, P. & Anderson, T. A. The Effects of Soil Organic Carbon Content on Plant Uptake of Soil Perfluoro Alkyl Acids (PFAAs) and the Potential Regulatory Implications. Environ Toxicol Chem 40, 820–833 (2021).

49. Lechner, M. & Knapp, H. Carryover of Perfluorooctanoic Acid (PFOA) and Perfluorooctane Sulfonate (PFOS) from Soil to Plant and Distribution to the Different Plant Compartments Studied in Cultures of Carrots (Daucus carota ssp Sativus), Potatoes (Solanum tuberosum), and Cucumbers (Cucumis Sativus). JOURNAL OF AGRICULTURAL AND FOOD CHEMISTRY 59, 11011–11018 (2011).

50. Lee, W.-Y., Iannucci-Berger, W. A., Eitzer, B. D., White, J. C. & Incorvia Mattina, M. Plant uptake and translocation of air-borne chlordane and comparison with the soil-to-plant route. Chemosphere 53, 111–121 (2003).

51. Lee, D.-Y., Choi, G.-H., Megson, D., Oh, K.-Y., Choi, I.-W., Seo, D.-C. & Kim, J.-H. Effect of soil organic matter on the plant uptake of perfluorooctanoic acid (PFOA) and perf luorooctanesulphonic acid (PFOS) in lettuce on granular activated carbon-applied soil. ENVIRONMENTAL GEOCHEMISTRY AND HEALTH 43, 2193–2202 (2021).

52. Liao, C., Liang, X., Lu, G., Thai, T., Xu, W. & Dang, Z. Effect of surfactant amendment to PAHs-contaminated soil for phytoremediation by maize (Zea mays L). Ecotox. Environ. Safe. 112, 1–6 (2015).

53. Lu, M.-X., Jiang, W. W., Wang, J.-L., Jian, Q., Shen, Y., Liu, X.-J. & Yu, X.-Y. Persistence and Dissipation of Chlorpyrifos in Brassica Chinensis, Lettuce, Celery, Asparagus Lettuce, Eggplant, and Pepper in a Greenhouse. PLoS One 9, e100556 (2014).

54. Mattina, M. J. I., Iannucci-Berger, W. & Dykas, L. Chlordane Uptake and Its Translocation in Food Crops. J. Agric. Food Chem. 48, 1909–1915 (2000).

55. Mattina, M. J. I., White, J., Eitzer, B. & Iannucci-Berger, W. Cycling of weathered chlordane residues in the environment: Compositional and chiral profiles in contiguous soil, vegetation, and air compartments. Environmental Toxicology and Chemistry 21, 281–288 (2002).

56. Matadha, N. Y., Mohapatra, S., Siddamallaiah, L., Udupi, V. R., Gadigeppa, S. & Raja, D. P. Uptake and distribution of fluopyram and tebuconazole residues in tomato and bell pepper plant tissues. Environ. Sci. Pollut. Res. 26, 6077–6086 (2019).

57. Mikes, O., Cupr, P., Trapp, S. & Klanova, J. Uptake of polychlorinated biphenyls and organochlorine pesticides from soil and air into radishes (Raphanus sativus). Environ. Pollut. 157, 488–496 (2009).

58. Müller, J. F., Hülster, A., Päpke, O., Ball, M. & Marschner, H. Transfer of PCDD/PCDF from contaminated soils into carrots, lettuce and peas. Chemosphere 29, 2175–2181 (1994).

59. Namiki, S., Otani, T., Motoki, Y., Seike, N. & Iwafune, T. Differential uptake and translocation of organic chemicals by several plant species from soil. J. Pestic. Sci. 43, 96–107 (2018).

60. Navarro, I., De La Torre, A., Sanz, P., Porcel, M. Á., Pro, J., Carbonell, G. & Martínez, M. D. L. Á. Uptake of perfluoroalkyl substances and halogenated flame retardants by crop plants grown in biosolids-amended soils. Environmental Research 152, 199–206 (2017).

61. Neuwirthova, N., Bilkova, Z., Vasickova, J., Hofman, J. & Bielska, L. Concentration/time-dependent dissipation, partitioning and plant accumulation of hazardous current-used pesticides and 2-hydroxyatrazine in sand and soil. Chemosphere 203, 219–227 (2018).

62. Pan, M. & Chu, L. M. Transfer of antibiotics from wastewater or animal manure to soil and edible crops. Environ. Pollut. 231, 829–836 (2017).

63. ADEME&INERIS. Etude des transferts de polluants organiques dans les plantes potagères en mettant en oeuvre une approche de terrain et une approche analytique. 1–181 (2003).

64. Saito, T., Otani, T., Seike, N. & Okazaki, M. A comparison of dieldrin residues in various vegetable crops cultivated in a contaminated field. Soil Science and Plant Nutrition 58, 373–383 (2012).

65. Samsøe-Petersen, L., Larsen, E. H., Larsen, P. B. & Bruun, P. Uptake of Trace Elements and PAHs by Fruit and Vegetables from Contaminated Soils. Environ. Sci. Technol. 36, 3057–3063 (2002).

66. Seike, N., Sakai, M., Murano, H., Okamoto, M., Saito, T., Narita, I., Hashimoto, Y., Ikeda, Y., Endo, M. & Otani, T. Relationship between dieldrin uptake in cucumber fruits and methanol–water extractable residue in soil. Journal of Pesticide Science 37, 252–257 (2012).

67. Shen, Y., Li, H., Ryser, E. T. & Zhang, W. Comparing root concentration factors of antibiotics for lettuce (Lactuca sativa) measured in rhizosphere and bulk soils. Chemosphere 262, 127677 (2021).

68. Shigei, M., Ahrens, L., Hazaymeh, A. & Dalahmeh, S. S. Per- and polyfluoroalkyl substances in water and soil in wastewater-irrigated farmland in Jordan. Science of The Total Environment 716, 137057 (2020).

69. Silvani, L., Hjartardottir, S., Bielska, L., Skulcova, L., Cornelissen, G., Nizzetto, L. & Hale, S. E. Can polyethylene passive samplers predict polychlorinated biphenyls (PCBs) uptake by earthworms and turnips in a biochar amended soil? Sci. Total Environ. 662, 873–880 (2019).

70.Singh, G., Kathpal, T., Spencer, W. & Dhankar, J. Dissipation of Some Organochlorine Insecticides in Cropped and Uncropped Soil. Environ. Pollut. 70, 219–239 (1991).

71. Stahl, T., Heyn, J., Thiele, H., Hüther, J., Failing, K., Georgii, S. & Brunn, H. Carryover of Perfluorooctanoic Acid (PFOA) and Perfluorooctane Sulfonate (PFOS) from Soil to Plants. Arch Environ Contam Toxicol 57, 289–298 (2009).

72. Sun, J., Wu, X. & Gan, J. Uptake and Metabolism of Phthalate Esters by Edible Plants. Environ. Sci. Technol. 49, 8471–8478 (2015).

73. Tang, Z., Huang, Q., Cheng, J., Qu, D., Yang, Y. & Guo, W. Distribution and accumulation of hexachlorobutadiene in soils and terrestrial organisms from an agricultural area, East China. Ecotox. Environ. Safe. 108, 329–334 (2014).

74. ADEME & INERIS. TROPHé - Transferts et Risques des Organiques Persistants pour l’Homme et les écosystèmes. La librairie ADEME at <https://librairie.ademe.fr/urbanisme-territoires-et-sols/1604-trophe-transferts-et-risques-des-organiques-persistants-pour-l-homme-et-les-ecosystemes.html>

75. Wang, Y., He, J., Wang, S., Luo, C., Yin, H. & Zhang, G. Characterisation and risk assessment of polycyclic aromatic hydrocarbons (PAHs) in soils and plants around e-waste dismantling sites in southern China. Environ. Sci. Pollut. Res. 24, 22173–22182 (2017).

76. Wang, Y., Li, X., Shen, J., Lang, H., Dong, S., Zhang, L., Fang, H. & Yu, Y. Uptake, translocation, and metabolism of thiamethoxam in soil by leek plants. Environ. Res. 211, 113084 (2022).

77. Wei, B., Liu, C., Bao, J., Wang, Y., Hu, J., Qi, M., Jin, J. & Wei, Y. Uptake and distributions of polycyclic aromatic hydrocarbons in cultivated plants around an E-waste disposal site in Southern China. Environ. Sci. Pollut. Res. 28, 2696–2706 (2021).

78. Wennrich, L., Popp, P. & Zeibig, M. Polycyclic Aromatic Hydrocarbon Burden in Fruit and Vegetable Species Cultivated in Allotments in an Industrial Area. International Journal of Environmental Analytical Chemistry 82, 667–690 (2002).

79. White, J. C., Mattina, M. I., Eitzer, B. D. & Iannucci-Berger, W. Tracking chlordane compositional and chiral profiles in soil and vegetation. Chemosphere 47, 639–646 (2002).

80. Wieczorek, J., Sienkiewicz, S., Pietrzak, M. & Wieczorek, Z. Uptake and phytotoxicity of anthracene and benzo[k]fluoranthene applied to the leaves of celery plants (Apium graveolens var. secalinum L.). Ecotox. Environ. Safe. 115, 19–25 (2015).

81. Witczak, A. & Abdel-Gawad, H. Comparison of organochlorine pesticides and polychlorinated biphenyls residues in vegetables, grain and soil from organic and conventional farming in Poland. Journal of Environmental Science and Health, Part B 47, 343–354 (2012).

82. Wu, X., Ding, Y., Wang, R., Rensing, C., Li, Y. & Feng, R. Differences in the uptake and bioconcentration of dichlorodiphenyltrichloroethane by eight vegetable cultivars and their health risk assessments. Chemosphere 215, 596–604 (2019).

83. Xiang, L., Chen, L., Yu, L.-Y., Yu, P.-F., Zhao, H.-M., Mo, C.-H., Li, Y.-W., Li, H., Cai, Q.-Y., Zhou, D.-M. & Wong, M.-H. Genotypic variation and mechanism in uptake and translocation of perfluorooctanoic acid (PFOA) in lettuce (Lactuca sativa L.) cultivars grown in PFOA-polluted soils. Science of The Total Environment 636, 999–1008 (2018).

84. Xu, Y., Du, W., Yin, Y., Sun, Y., Ji, R., He, H., Yang, S., Li, S., Wu, J. & Guo, H. CuO nanoparticles modify bioaccumulation of perfluorooctanoic acid in radish (Raphanus sativus L.). Environmental Pollutants and Bioavailability 34, 34–41 (2022).

85. Ye, M., Sun, M., Feng, Y., Li, X., Schwab, A. P., Wan, J., Liu, M., Tian, D., Liu, K., Wu, J. & Jiang, X. Calcined Eggshell Waste for Mitigating Soil Antibiotic-Resistant Bacteria/Antibiotic Resistance Gene Dissemination and Accumulation in Bell Pepper. J. Agric. Food Chem. 64, 5446–5453 (2016).

86. Yin, R., Lin, X. G., Wang, S. G. & Zhang, H. Y. Effect of DBP/DEHP in vegetable planted soil on the quality of capsicum fruit. Chemosphere 50, 801–805 (2003).

87. Yu, X., Liu, X., Liu, H., Chen, J. & Sun, Y. The accumulation and distribution of five antibiotics from soil in 12 cultivars of pak choi. Environ. Pollut. 254, 113115 (2019).

88. Yuan, L., Cheng, J., Wang, Y., Liu, Y., Wang, W., Gao, R. & Yu, X.-Y. Uptake and toxicity of di-(2-ethylhexyl) phthalate in Brassica chinensis L. Chemosphere 252, 126640 (2020).

89. Zhang, M., Liang, Y., Son, A., Yu, B., Zeng, X., Chen, M.-S., Yin, H., Zhang, X., Sun, B. & Fan, F. Loss of soil microbial diversity may increase insecticide uptake by crop. Agric. Ecosyst. Environ. 240, 84–91 (2017).

90. Zohair, A., Salim, A.-B., Soyibo, A. A. & Beck, A. J. Residues of polycyclic aromatic hydrocarbons (PAHs), polychlorinated biphenyls (PCBs) and organochlorine pesticides in organically-farmed vegetables. Chemosphere 63, 541–553 (2006).
